# Synaptic vesicle capture by CaV2.2 calcium channels

**DOI:** 10.3389/fncel.2013.00101

**Published:** 2013-06-28

**Authors:** Fiona K. Wong, Qi Li, Elise F. Stanley

**Affiliations:** Laboratory of Synaptic Transmission, Genetics and Development Division, Toronto Western Research InstituteToronto, ON, Canada

**Keywords:** presynaptic, calcium channel, synaptic vesicle, tethering, docking, SV-PD, CaV2.2, transmitter release

## Abstract

The fusion of synaptic vesicles (SVs) at the presynaptic transmitter release face is gated by Ca^2^^+^ influx from nearby voltage-gated calcium channels (CaVs). Functional studies favor a direct molecular “tethering” attachment and recent studies have proposed a direct link to the channel C-terminal. To test for direct CaV–SV attachment we developed an *in vitro* assay, termed SV pull-down (SV-PD), to test for capture of purified, intact SVs. Antibody-immobilized presynaptic or expressed CaV2.2 channels but not plain beads, IgG or pre-blocked antibody successfully captured SVs, as assessed byWestern blot for a variety of protein markers. SV-PD was also observed with terminal fusion proteins of the distal half of the C-terminal, supporting involvement of this CaV region in tethering. Thus our results support a model in which the SV tethers directly to the CaV. Since the tip of the C-terminal could extend as far as 200 nm into the cytoplasm, we hypothesize that this link may serve as the initial SV capture mechanism by the release site. Further studies will be necessary to evaluate the molecular basis of C-terminal tethering and whether the SV binds to the channel by additional, shorter-range attachments.

## INTRODUCTION

The general sequence of processes that precede release of transmitters from presynaptic terminals at fast synapses is well established: a transmitter filled vesicle that docks to the membrane is gated to fuse and discharge its contents by the influx of Ca^2^^+^ ions through voltage-gated calcium channels (CaVs). It is now generally accepted that the relationship between these two elements is very intimate (Stanley, 1997; Mulligan et al., 2001; Lisman et al., 2007; Bucurenciu et al., 2008; Eggermann et al., 2011; Schmidt et al., 2013). The finding that a single channel can gate fusion localized the synaptic vesicle (SV) to individual CaV Ca^2^^+^ domains provided the first functional evidence for SV “tethering” (Stanley, 1993) and a putative scaffold-like structure, linking presynaptic calcium channels to the SV, has been imaged by electron microscope (EM) tomography (Harlow et al., 2001).

**FIGURE 1:**
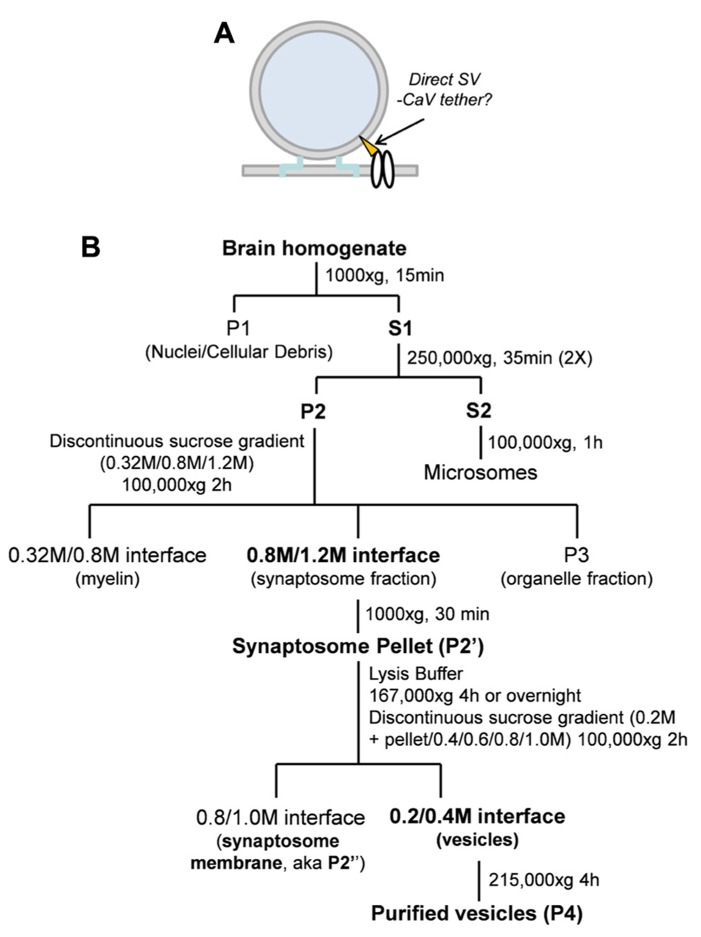
** (A)** Hypothetical model of SV tethering. The SV is tethered directly to the calcium channel. **(B)** Synaptosome membrane and synaptic vesicle purification protocols (see Materials and Methods).

The simplest mechanism to ensure close SV and channel association is via a direct molecular tether (**Figure 1A**) and a variety of proteins that might contribute to channel-SV tethering have been reported (Saisu et al., 1991; Sheng et al., 1994; Coppola et al., 2001; Kiyonaka et al., 2007; Kaeser et al., 2011). A current hypothesis is that the SV can bind directly to the channel and that this is via a link to the distal tip of the channel C-terminal (Kaeser et al., 2012). However, this concept is based on indirect observations, such as yeast two-hybrid analysis of protein interactions, and has never been tested directly. Our primary object was to test if SVs can bind directly to calcium channels. We used chick brain as the experimental material and devised a cell-free assay in which we tested if immobilized presynaptic CaV2.2 calcium channels (the predominant presynaptic CaV in chick; Stanley and Atrakchi, 1990; Stanley, 1991; Gruner and Silva, 1994; Sivaramakrishnan and Laurent, 1995) could capture sucrose gradient-purified chick brain SVs *in vitro*. We tested for SV capture by standard Western blot for signature vesicle proteins and report the first direct evidence of channel-SV binding. The idea that the SV can bind to the distal C-terminal was tested by using the same SV capture method but with a C-terminal fusion protein as the bait.

## MATERIALS AND METHODS

### DISSECTION AND PREPARATION OF FRESH BRAIN FRACTIONS

All chemicals were from Sigma-Aldrich Co. unless otherwise noted. Embryonic chick brains were dissected and fractionated as described (Huttner et al., 1995; Juhaszova et al., 2000; Wong and Stanley, 2010) with minor modifications (**Figure 1B**). Chick brains ranging from E15 to E17 were removed and washed quickly in an ice-cold sucrose-based homogenization buffer [(HB; 0.32 M sucrose, 10 mM 4-(2-hydroxyethyl)-1-piperazineethanesulfonic acid (HEPES) pH 7.4, 2 mM ethylenediaminetetraacetic acid (EDTA)] and homogenized using 10 strokes with a glass homogenizer (Thomas Scientific) in HB supplemented with 1 mM phenylmethanesulfonylfluoride (PMSF; Roche) and protease inhibitor cocktail. Homogenized brains were then spun at 1000 × *g* to pellet nuclear and cellular debris. The resulting supernatant (S1) was pooled and spun in a Beckman ultracentrifuge at 250,000 × *g* (Type 70 Ti rotor; all rotors were Beckman) for 35 min to pellet (P2). P2 was resuspended in HB to wash and the spin was repeated. P2 was loaded onto a differential sucrose gradient 0.32 M (sample)/0.8/1.2 M (sucrose) and centrifuged at 100,000 × *g* (SW41 rotor) for 1.5 h and without a brake during deceleration.

Synaptosomes were isolated from the 0.8/1.2 M sucrose interface and spun at 20,000 × *g* (Type 70Ti rotor) and washed in HB to remove sucrose. The synaptosomes were lysed by osmotic shock with a HEPES-based lysis buffer (50 mM HEPES pH 7.4, 2 mM EDTA, supplemented with 1 mM PMSF and protease inhibitor cocktail) and centrifuged at 165,000 × *g* (Type 70Ti rotor) for 4 h or overnight. The resulting pellet, P2’, was resuspended in 0.2 M HEPES-buffered sucrose and loaded onto a discontinuous sucrose gradient (sample/0.4/0.6/0.8/1.0 M sucrose) and centrifuged at 100,000 × *g* (SW41 rotor) for 1.5 h without braking. Enrichment of synaptosomes was demonstrated by Western blot which showed retention of surface membrane marker proteins (CaV2.2, Na/K ATPase) and SV proteins [(synaptotagmin-1 (STG1), VAMP (vesicle associated protein-2)] with exclusion of markers for Golgi (GM130) and endosomes (early endosome marker-1, EEA1; **Figure 3B**).

Vesicles were isolated from the 0.2 M/0.4 M layer interface, diluted in 0.1 M HEPES-buffered sucrose and pelleted at 215,000 × *g* (SW60 rotor). A presynaptic membrane-enriched fraction (synaptosomes are composed of presynaptic nerve terminal together with an attached “scab” of the postsynaptic apparatus), and termed “synaptosome surface membrane,” was isolated from the 0.8/1.0 M interface of the same spin and was washed by dilution in HB and re-centrifuged.

Purified vesicles (P4) were resuspended in HB or modified radioimmunoprecipitation assay (RIPA) buffer (50 mM Tris–HCl pH 7.4, 1 mM EDTA, 150 mM NaCl, 1% NP-40, 0.5% Na deoxycholate; supplemented with 1 mM PMSF and protease inhibitor cocktail; herein RIPA) and membranes (P2″) were solubilized in RIPA buffer and passaged 3× in a 30½ G syringe before use in experiments. Concentrations of brain fractions were determined using the Bradford concentration assay (Bradford reagent) and DU640 spectrophotometer (Beckman Coulter). Varying concentrations of bovine serum albumin (BSA) were used as standards and standard curves were plotted before determining the approximate concentration of the samples.

### CaV2.2 EXPRESSION

CaV2.2 channels were expressed as described (Chan et al., 2007). Briefly, plasmids encoding rat CaV2.2 subunits (α1B, α2δ, and β1b) in pMT2 vector for expression in mammalian cell lines were all kindly provided by Dr. T. Snutch (University of British Columbia). tSA201 cell lines (kindly provided by Dr. L. C. Schlichter, Toronto Western Research Institute) were transfected with CaV subunits using Lipofectamine 2000 according to manufacturer’s instructions in Dulbecco’s modified Eagle’s medium (DMEM) medium (Invitrogen). DMEM was removed approximately 4–5 h after transfection and replaced with DMEM containing 1% penicillin–streptomycin (Invitrogen) and 10% fetal bovine serum (Invitrogen). Approximately 48 h after transfection, the cells were collected and solubilized in RIPA buffer supplemented with 1 mM PMSF and protease inhibitor cocktail.

### WESTERN BLOT

Immunoprecipitation (IP) complexes were washed with either HB (intact vesicles) or RIPA buffer (solubilized vesicles or membranes) five times before adding 4× Laemmli sample buffer (Bio-Rad) with 5% β-mercaptoethanol, and boiled for 5 min at 100°C to denature proteins. Samples were placed on ice to stop the reaction and spun quickly to pellet IP complexes. Approximately 10 μl of Precision Plus kaleidoscope protein ladder (Bio-Rad) or 1.5–2 μg sample was loaded onto each lane of 8%/12% acrylamide (Sigma) step gradient separating gels with a 4% acrylamide stacking gel for sodium dodecyl sulfate polyacrylamide gel electrophoresis [SDS-PAGE; all Bio-Rad except for tetramethylethylenediamine (TEMED) which is Bioshop]. Proteins were transferred onto Immobilon-P polyvinylidene fluoride (PVDF) membranes (Bio-Rad) and blocked for 1 h with 5% skim milk (Bioshop) in 1× TBS-T (10 mM Tris–HCl pH 8.0, 150 mM NaCl, 0.1% Tween-20; all Bioshop). Membranes were immunoblotted for 2 h at room temperature or overnight at 4°C in 5% milk in TBS-T. Blots were washed three times with TBS-T for 10 min each and then incubated with goat anti-mouse or goat anti-rabbit secondary antibody conjugated to horseradish peroxidase (1:3000; Jackson ImmunoResearch) in 5% milk in TBS-T for 1 h at room temperature. Blots were washed three times with TBS-T for 10 min each and treated with enhanced chemiluminescence reagent (Amersham Biosciences) for 3 min before exposure to film (Denville Scientific Inc.). Films were developed using the Konica SRX-101A developer and scanned as JPEG images using a Canon Canoscan LiDE 25 Scanner. Western blots of the starting material loaded a high 4%, and/or low 1.5% fraction of protein input, labeled as SV (h) and SV (l), respectively.

### ANTIBODIES AND OTHER MATERIALS

Antibodies used in this study are listed in **Table 1**. Ab571, the primary antibody used in this study, was raised against chick CaV2.2 and has been characterized extensively (Li et al., 2004) including identification of the captured target channel by mass spectroscopy (Gardezi et al., 2010). L4569 has also been demonstrated to identify the long-splice, release site-associated, variant of the channel (Khanna et al., 2006b). Inevitably, commercial polyclonal Abs often exhibited variability between batches, affecting the band patterns and target protein-detection reliability, a problem that was noted in particular for anti-VAMP (see below).

**Table 1:** Antibodies used in this study.

Antibody	Target	Poly/mono	Source	IP dilution	WB dilution
Ab571	Cav2.2 II–III loop	P	E. F. Stanley (Li et al., 2004)	1:400	1:2000
EEA1	Early endosome	M	BD Bioscience	–	1:2000
FLAG	FLAG	P	Cell Signalling Technology	–	1:4000
GM130	Golgi	P	Sigma-Aldrich Co.	–	1:2000
HPC-1	Syntaxin 1A	M	Sigma-Aldrich Co.	–	1:5000
L4569 (Cav2.2)	Cav2.2 distal C-terminal	P	E. F. Stanley (Khanna et al., 2006b)	–	1:2000
Munc18	Munc 18-1	P	ABR	–	1:2000
Na/K ATPase	Na/K ATPase	M	Santa-Cruz Biotechnology	–	1:1000
Rab3a	Rab3a	M	Synaptic Systems GMBH	–	1:1000
RIM1	RIM (non-specific)	M	BD Biosciences	–	1:1000
RIM1a	RIM (RIM1 specific)	P	Synaptic Systems GMBH	–	1:2000
RIM2	RIM (non-specific)	P	Synaptic Systems GMBH	–	1:2000
SV43574	SNAP25	M	Sternberger Monoclonals Inc.	–	1:2000
Strep	Strep	M	Sigma-Aldrich Co.	–	1:1000
SV2A	SV2A	M	Synaptic Systems GMBH	–	1:1000
SV2A	SV2A	P	Synaptic Systems GMBH	1:400	1:2000
ASV30	Synaptotagmin	M	Abcam Inc.	1:400	1:1000
Synapsin 1	Synapsin	P	Synaptic Systems GMBH	–	1:1000
VAMP2 (EMD)	VAMP2	P	EMD Chemicals	–	1:1000
VAMP2 (Enzo)	VAMP2	P	Enzo Life Sciences	–	1:1000

### ANTIBODY IMMOBILIZATION AND CaV2.2 CAPTURE

Antibodies (see **Table 1**) were immobilized to either protein A agarose (for rabbit polyclonal antibodies) or protein G sepharose beads (for mouse monoclonal antibodies) for at least 4 h in 1× phosphate buffered saline (PBS; Gibco). Antibody–bead complexes were washed four times with PBS and then incubated overnight on a rotator at 4°C with solubilized presynaptic membranes (IP) already pre-cleared for 1 h with protein A/G beads to capture proteins. CaV2.2 was immobilized as above (see also Li et al., 2004; Wong and Stanley, 2010). Briefly, Ab571 was incubated in 1× PBS (Gibco) with protein A agarose beads at 4°C for 4 h or overnight. The antibody–bead complexes were washed four times with PBS, incubated for 15 min with high salt (RIPA + 1.15 M NaCl; Khanna et al., 2006a), and washed four times with RIPA buffer prior to incubation with purified synaptosome membrane lysates (see Results). Several standard controls were used, including plain beads and pre-immune antibody. In addition, we pre-blocked Ab571 with the peptide used for its immunopurification (3 mM, for 2 h at 4°C) before washing and brain fraction incubation.

### GENERATION OF FUSION PROTEINS

Chick E15 brain mRNA was used to synthesize cDNA using the reverse transcriptase II enzyme (Invitrogen). cDNA was used as a template for reverse transcription polymerase chain reaction (RT-PCR). For C3_strep_ a PCR fragment of the CaV2.2 long-splice (cdb1) variant (aa 2138–2357) was inserted into the TA cloning vector pBR2.1 (Invitrogen) and cut out at *Eco*RI and *Xho*I sites, then subcloned into pPr-IBA (IBA) expression vector. The DNA sequence in frame was confirmed by sequencing after transformation into DH5α competent cells (Invitrogen). Constructs were transformed into BL21 (DE3; Invitrogen) for fusion protein production.

### FUSION PROTEIN PURIFICATION

Fusion proteins were grown and purified using standard protocols. C3_s__trep_ bacteria pellets were resuspended in lysis buffer (0.1 M Tris pH 8.0, 150 mM NaCl, 1 mM EDTA, 0.1% Triton X-100, 1 mM PMSF and protease inhibitors) before sonication using 20 bursts for 5–10 s each and incubation on ice for 30 min. Lysates were then vortexed, incubated on ice for 30 min and centrifuged. The supernatant was incubated with strep-tactin superflow beads (IBA) for 2 h on ice and washed three times with lysis buffer. Protein was eluted with elution buffer (0.1 M Tris pH 8.0, 150 mM NaCl, 1 mM EDTA, 0.1% Triton X-100, 1 mM PMSF plus 10 mM D-desthiobiotin), collected and injected into a Slide-A-Lyzer 10 K dialysis cassette (Pierce) and dialyzed against PBS with 0.05% Triton X-100 and 1 mM PMSF at 4 C for 24 h. Protein yield was analyzed using SDS-PAGE and Coomassie blue gel staining by comparison with BSA standards.

### INTERACTION ASSAYS

**FIGURE 2:**
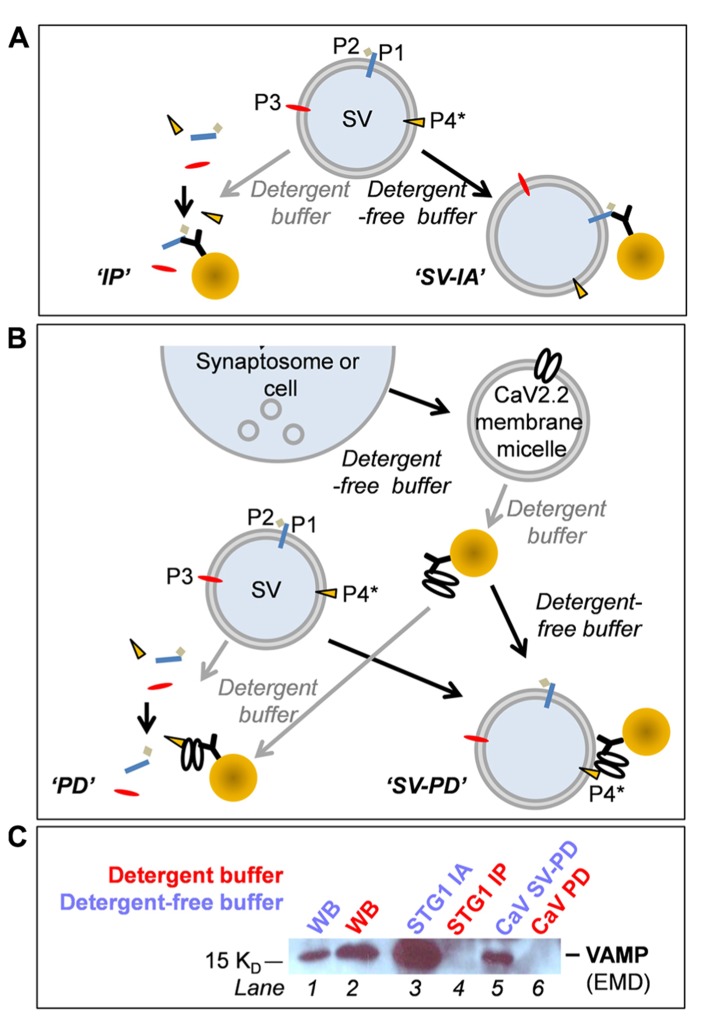
** (A–C)** Biochemical methods (see text). **(A)** Immunoprecipitation (IP) and SV-immunoadsorption (SV-IA). P1, P2, P3, and P4 are hypothetical SV proteins. P1, P3, and P4 are integral vesicle proteins while P2 is a vesicle-associated protein (bound to an integral protein). P4 is also the putative tether attachment protein, as flagged by the asterisk. **(B)** Protein pull-down (PD) and whole SV pull-down (SV-PD). **(C)** The four methods compared using the SV protein, VAMP as the SV marker. Lanes 1 and 2: Western blot lanes demonstrate VAMP in the “detergent-free” (HB) and “detergent-containing” (RIPA buffer) samples adding ~4% of the sample used for IPs. Lane 3: VAMP was detected after SVs were captured by SV-IA using anti-STG1. Lane 4: Anti-STG1 failed to capture VAMP from solubilized SVs because these two proteins are not direct binding partners. Lane 5: CaV2.2 channels were immobilized from synaptosome membrane lysate and incubated with a suspension of SVs and pull-down VAMP. Lane 6: CaV2.2 channels failed to capture VAMP from solubilized SVs.

All assays utilized purified SVs as the starting material either solubilized in detergent-containing RIPA buffer or suspended intact in HB. These SV lysates and SV suspensions were used in four main biochemical assays as illustrated (**Figures 2A–C**).

**FIGURE 3:**
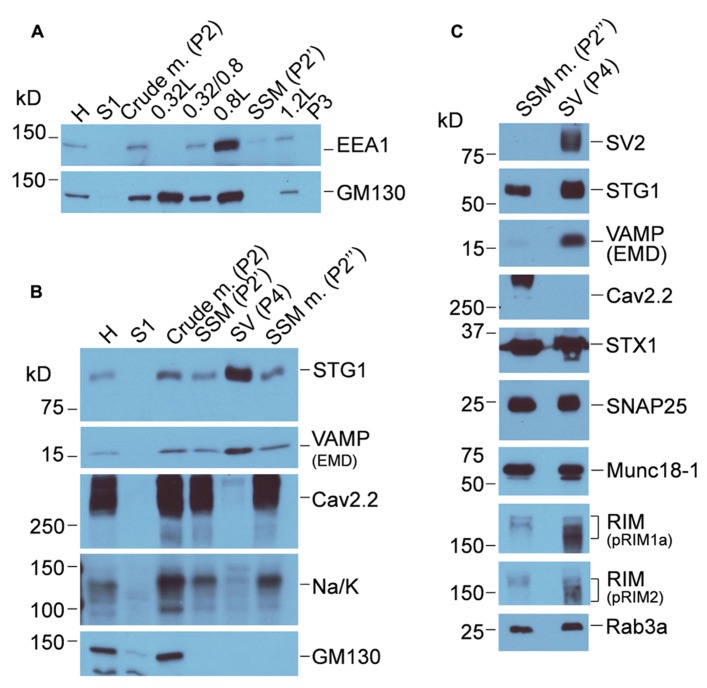
** Characterization of chick brain fractions.** **(A)** Western blot of fractions from the first sucrose gradient probed for EEA1 (EEA1, early endosome marker 1) and (GM130 Golgi matrix protein 130), markers of endosome and Golgi membranes, respectively. Crude m., crude brain membranes; SSM, synaptosome. **(B)** Sucrose gradient fractions probed for synaptic vesicle (STG1, VAMP), Golgi (GM130) and two surface membrane makers, CaV2.2 and Na/K (Na/K ATPase). SSM m., synaptosome membrane; SV, synaptic vesicle. **(C)** Western blots comparing proteins in the synaptosome surface membrane and SV fractions. The same protein load was added to each lane. Purity of the SV fraction is indicated by the enrichment of SV2 and VAMP. However, many proteins generally associated with one or other compartment were present in both, including Rab3a, STG1, synapsin, STX1, and SNAP25. CaV2.2 serves as a surface membrane marker. See **Figure 1** for the fraction origins.

In the first pair of methods (**Figure 2A**) an antibody against an SV protein was immobilized on precipitation beads. These were then used either to capture the protein plus its binding partners from solubilized SVs, by standard pull-down (IP; **Figure 2A**, left), or to capture the entire SV from the suspension by immunoadsorption (IA; **Figure 2A**, right).

We combined pull-down (PD) with IA to as an assay for SV tethering, using CaV2.2 itself as a “bait” (**Figure 2B**, right). The channel was first captured by IP from solubilized synaptosome membrane (RIPA) with immobilized Ab571. The beads were then washed thoroughly (*as above*) in RIPA buffer with high salt (1.15 M NaCl) to shed associated proteins (Khanna et al., 2006a) and then in HB to remove any detergent. Immobilized CaV2.2 were then incubated with suspended SVs and SV capture was assayed by WB using several protein markers. This is in essence a “synaptic vesicle pull-down” method from which we derive the term SV-PD. It is also possible to incubate the immobilized bait with solubilized SVs to test for direct protein binding (**Figure 2B**, left); a more standard PD approach using an antibody-immobilized bait.

Anti-STG1 or immobilized CaV2.2 was used to contrast the outcome of these methods in a single blot with VAMP as the SV marker (**Figure 2C**). Since STG1 and VAMP are not binding partners, anti-STG1 antibody failed to co-IP VAMP from solubilized SVs. However, anti-STG1 captured VAMP by IA as part of the intact SV. Similarly, immobilized CaV2.2 did not recover VAMP from an SV lysate but did as part of the intact SV. These results are discussed in detail below.

### QUANTITATIVE ANALYSIS

Blots were exposed to film for varying durations ranging from a few seconds to overnight permitting us to select exposures where the band for the protein of interest was within the dynamic range of the film, as assessed visually by the absence of band saturation. Using the gel scanning program UN-SCAN-IT (Silk Scientific Inc.) we measured the intensities of the protein (*x*), the corresponding control (*c*), and the Western blot [*wb*, using the vesicle (l) lane] band intensities. The scanning program reads an unstained region of the blot to correct for background and yield the specific signal intensities *x*_i_, *c*_i_, and *wb*_i_. Experiments in which the intensity of the control lane (strep vector) was more than 50% of the vesicle (l) lane were not analyzed further. We determined *X*, the captured protein intensity as: *X* = *x*_i_ - *c*_i_. Finally, to permit comparison of captured protein intensities between different experiments, we normalized these values to *wb*_i_ to give us *X*_norm_: *X*_nor__m_ = *X*/*wb*_i_. [In experiments without a SV(l) lane we estimated the *wb*_i_ value from the mean SV(l)/SV(h) value for that protein]. The normalized values were used to calculate the mean (${\overset{¯}{X}}_{norm}$) and standard error (SE) for each measurement (in dimensionless units, U), omitting any values that were ${\overset{¯}{X}}_{norm}$ ± (2 × SD). Significant PD was tested using a one-sample *t*-test (*p*_t = 0_) with the null hypothesis that ${\overset{¯}{X}}_{norm}$ = 0, with *p*_t = 0_ < 0.05 considered significant.

## RESULTS

### SYNAPTIC VESICLE PROTEIN MARKERS

Western blot was used to confirm purification of synaptosomes from other membrane compartments including endosomes, Golgi (**Figure 3A**) endoplasmic reticulum (using anti-calnexin, *data not shown*) and purification of the synaptosome membrane and SV fractions after the second sucrose gradient (**Figures 3B,C**). The virtually complete exclusion of Na/K ATPase and the CaV2.2 itself from the SVs fraction confirms the enrichment of the latter fraction by the second sucrose gradient centrifugation step, consistent with numerous previous studies (e.g., Huttner et al., 1995; Juhaszova et al., 2000; Wong and Stanley, 2010). The purity of the SVs is supported by the enrichment of key marker proteins such as SV2, VAMP, and Rab3a interacting molecule (RIM). Canonical SV proteins can also reside in the nerve terminal surface membrane consistent with their exchange during the SV cycle, and some, such as STG1 (Khanna et al., 2006a), appear to be invariably present. It should be noted that the soluble *N*-ethylmaleimide-sensitive factor (NSF) attachment protein receptor (SNARE) proteins syntaxin 1 (STX1) and synaptosomal-associated protein of 25 kDa (SNAP25), which are generally regarded as surface membrane components, are also present also in SVs (Walch-Solimena et al., 1995; Takamori et al., 2006) and can serve as SV markers.

### NATIVE CaV2.2 CAN CAPTURE INTACT SVs

We first tested if CaV2.2 channels could capture SVs *in vitro* by SV-PD (see **Figure 2B**, right). This required combined IP/PD experiments and were technically challenging. After discovering that freeze/thaw resulted in a reduced repeatability, all experiments were carried out on fresh brain fractions, limiting us to about one experiment a week. Some non-specific binding, evident as protein bands on the control beads, is to be expected with detergent-free buffer experiments but could not be detected until the final blots were viewed. An experiment was deemed acceptable when positive control test bands were unquestionably darker than controls to the unaided eye; where this was not the case the experiment was discarded.

**FIGURE 4:**
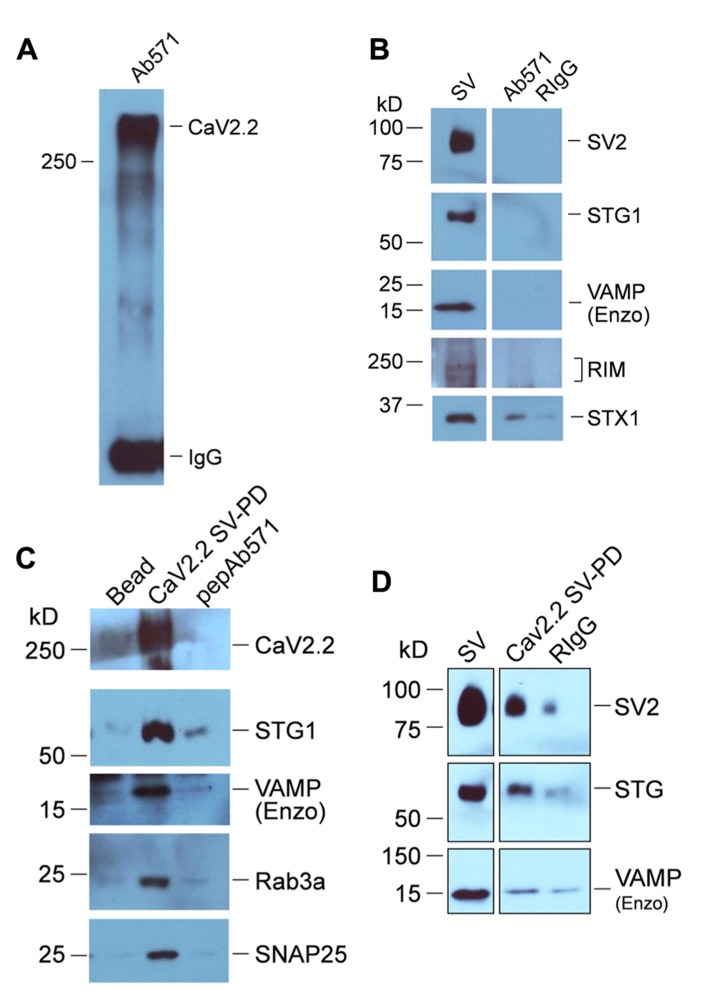
** SV-PD by synaptosome membrane CaV2.2.** **(A)** CaV2.2 channels were captured by standard IP from synaptosome surface membrane lysate using Ab571. Note: the band for this large ~250 kD protein often runs at a higher molecular weight. The IgG 50 kD band is also shown. **(B)** CaV2.2 IP’d by Ab571, as in **(A)** was tested for SV marker proteins by western blot. Most markers (SV2, STG1, and VAMP are shown) were negative except STX1 (see text). **(C,D)** Two examples of SV capture by CaV2.2 from separate experiments. The channel was immobilized from synaptic membrane lysate as in **(A)** and was then washed in detergent-free HB prior to incubation with a suspension of chick SVs (purified as above). Controls were untreated beads (*Beads*) or Ab571 pre-blocked with its immunoprecipitation peptide (*pepAb571*, *N* = 3) in **(C)** and pre-immune rabbit IgG (*RIgG*) in **(D)** After rinsing in HB, captured proteins were assessed byWB. Capture of SVs by only beads with pre-bound CaV2.2 (*CaV2.2 SV-PD lanes*) was indicated by the recovery of multiple protein markers. The number of experiments carried out for SV protein Cav2.2 SV-PD was *N*_STG1_ = 10; *N*_SV2_ = 6; *N*_RIM_ = 6.

We first immobilized CaV2.2. To ensure that the channels would be typical of presynaptic terminals, which are known to include the long C-terminal splice variant (Maximov and Bezprozvanny, 2002; Khanna et al., 2006b), we used freshly prepared synaptosome membranes as the CaV2.2 source. These were solubilized and CaV2.2 was captured by standard IP (**Figure 4A**). Since presynaptic CaV2.2 co-IPs with a number of release site-associated proteins (Sheng et al., 1998; Khanna et al., 2007) which might interfere with the SV-PD assay, the CaV2.2-beads were rinsed thoroughly in a high salt wash to facilitate shedding of weakly associated binding partners (Khanna et al., 2006a). After rewashing in HB, immobilized CaV2.2 were virtually free of SV-associated proteins, as assessed by WB (**Figure 4B**; but note a faint recovery of STX1 with CaV2.2, consistent with previous reports; Saisu et al., 1991; Sheng et al., 1994; Li et al., 2004) and were incubated with the SV suspension to permit SV capture. The beads were rewashed and analyzed for captured proteins by standard WB. A number of SV marker proteins were recovered by the immobilized CaV2.2 (**Figures 4C,D**), including STG [0.41 ± (SE)0.13 U, *N* = 10; *p*_t = 0_ < 0.01) and SV2 (0.27 ± 0.06 U, *N* = 6; *p*_t = 0_ < 0.05) consistent with captured of the intact SV (SV-PD). The mean normalized band intensity for RIM was much higher but failed to reach significance due to a greater variability (0.930 ± 0.396 U, *N* = 6; *p*_t = 0_ = 0.065). This interpretation was supported by negative results with controls: plain IP beads or immobilized Ab571 pre-blocked with its affinity-purification peptide (Li et al., 2004) failed to SV-PD (*N* = 3; **Figure 4C**). Thus, we conclude that presynaptic terminal CaV2.2 immobilized on beads can capture SVs *in vitro*.

### DOES CaV2.2 REQUIRE SYNAPTOSOME FACTORS TO LINK TO PURIFIED SVs?

**FIGURE 5:**
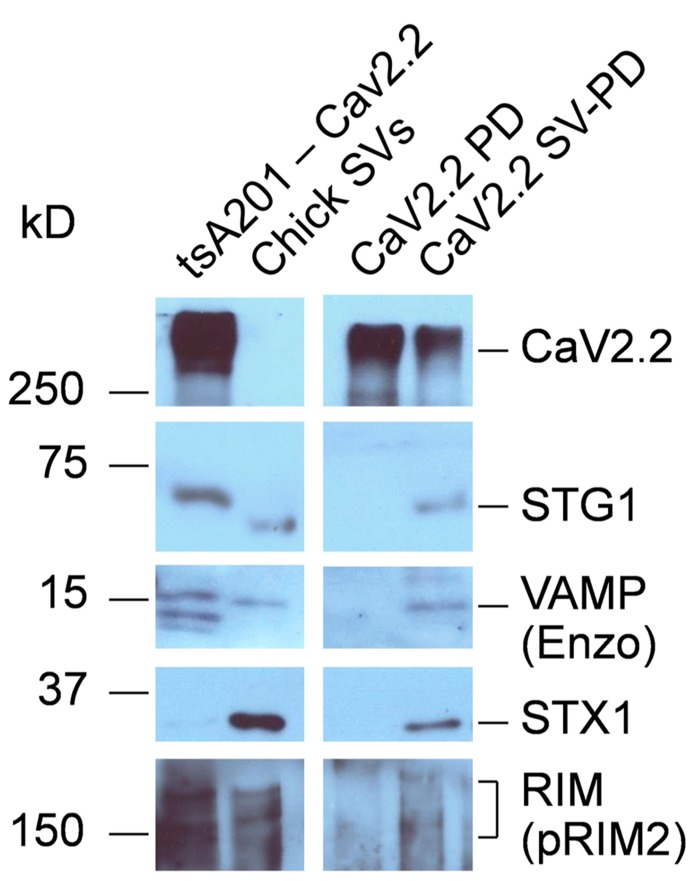
** SV-PD by expressed CaV2.2.** CaV2.2 channels were expressed in tsA201 cells. Western blot of these cells exhibited bands corresponding to the channel and also SV protein proteins *TsA201-CaV2.2*. The cells were solubilized in RIPA buffer and their CaV2.2 channels were captured on IP beads, as described. The immobilized CaV2.2 was then incubated either with either solubilized (*CaV2.2 PD*) or intact (*CaV2.2 SV-PD*) SVs, purified from chick brain as described. With the exception of a faint band for RIM (*contrast enhanced*), no SV proteins were co-precipitated using the solubilized SVs (*CaV2.2 PD*), and hence, these proteins were neither retained from the original cell lysate nor bind to the channel directly from the solubilized SVs. However, marker proteins were captured when the beads were exposed to intact SVs (*CaV2.2 SV-PD*), consistent with capture of the entire SV, even though the lighter CaV2.2 band indicating that somewhat less IP beads were used. The un-evenness in the STG1 band reflects a minor discontinuity in the gel (the antibody is highly selective). STX1 serves here as an additional SV marker (as in **Figure 3**). Similar results were observed in three separate experiments.

The possibility remained that, despite the high salt wash, the presynaptic CaV2.2 channels were co-purified with a presynaptic-specific protein that acts as an adaptor to the SV. To gain insight into this question we expressed CaV2.2 α1B in tsA201 cells (with a CaVβ and α_2_-δ subunits that are necessary for efficient expression) as previously described (Chan et al., 2007). The cells were solubilized and the channels captured by IP as above for use in the SV-PD and PD assays. The expressed immobilized CaV2.2 failed to PD SV proteins from the RIPA solubilized SVs, but SV protein markers were effectively recovered with intact SVs suspended in the HB buffer (*N* = 3; **Figure 5**), consistent with recovery of the intact SV. Thus, CaV2.2 derived from a non-presynaptic source can capture SV. To some extent expression of RIM in these cells varied with batches. Thus, faint protein bands were seen in these experiments (**Figure 5**) but were almost undetectable in a later batch (Gardezi et al., 2013).

### CAN SVs BE CAPTURED BY THE CHANNEL C-TERMINAL?

**FIGURE 6:**
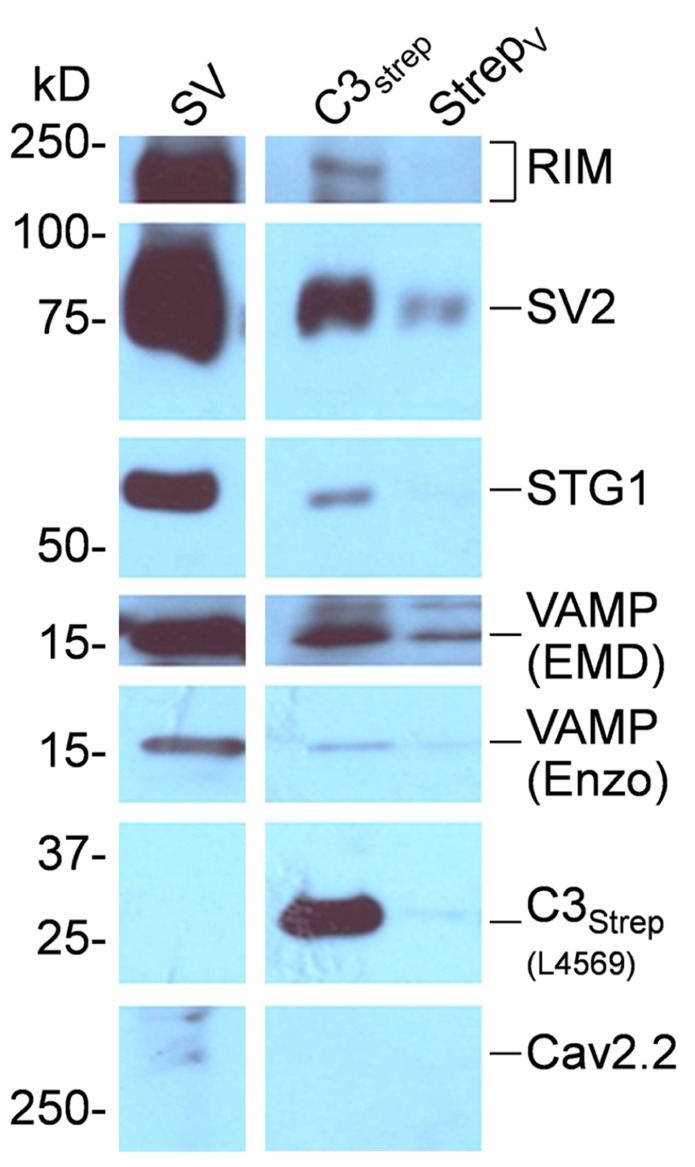
** SV capture by the CaV2.2 distal C-terminal.** C3_strep_ was immobilized on beads and used for SV-PD. The fusion protein is identified on theWB by L4569, an antibody raised against the distal end of the long-splice C-terminal region, but is negative for the control expressed vector lane (Strep_*V*_). C3_strep_ SV-PD was evident from the much darker SV protein bands than observed in the control lane. C3_s__trep_ SV-PD: *N*_RIM_ = 9; *N*_STG1_ = 6; *N*_SV2_ = 7. In this example a trace amount of CaV2.2 was observed in the SV fraction.

To test for direct binding to the calcium channel we created an One Strep-tagged C-terminal fusion protein, C3_strep_, covering the distal half of the C-terminal to its tip (see Gardezi et al., 2013). SV-PD was carried out by incubating bead-immobilized C3_strep_ with SVs suspended in detergent-free buffer, using expressed vector as a control. After wash, attached proteins were denatured and assayed by WB, as above. Recovery of a number of SV proteins, including RIM (2.96 ± 0.63 U, *N* = 9; *p*_t__=__0_ = <0.01), STG1 (0.25 ± 0.06 U, *N* = 6; *p*_t__=__0_ = <0.01), and SV2 (0.28 ± 0.09 U, *N* = 7; *p*_t__=__0_ = <0.05), confirmed capture of the SV (**Figure 6**).

## DISCUSSION

The main finding in this report is that SVs can be captured by intact CaV2.2 channels, either isolated from presynaptic terminals or expressed in a mammalian cell line, and this capture can be replicated using a distal C-terminal fusion protein. These findings support the hypotheses that SVs scaffold directly to the channel (**Figure 1**) and that the scaffolding can occur via the channel C-terminal.

The *in vitro* assays used in this study require presynaptic membrane and SV fractions from fresh brain tissue. The former was used solely as the source material for the immobilization of CaV2.2 channels, the purity of which was ensured with a highly characterized specific antibody, Ab571 (Li et al., 2004; Gardezi et al., 2010). We monitored the purity of SVs by Western blot of corresponding fractions and demonstrate marked enrichment. Each SV capture experiment was assayed using three or more SV protein markers to ensure that the assay reflected capture of SVs. It remains possible, however, that other cytoplasmic vesicles or fragments, that include at least one of the markers and that were co-captured with CaV2.2 contributed to the protein bands. Dense core vesicles (DCVs) are one possibility. While intact DCVs partition to a deeper region of the gradient the SV fraction may contain DCV membrane fragments (Walch-Solimena et al., 1993) that could be captured and could contribute to the STG1 protein band (Walch-Solimena et al., 1993). In any case, the combination of a starting material that is highly enriched for SVs, together with the multi SV marker protein-detection approach is entirely consistent with the capture of intact SVs by both CaV2.2 and its C-terminal fusion protein.

The method developed in this report is based on two predecessors: the first is IA which was developed specifically to purify lipid vesicles from cell membrane fractions, and second, the pull-down method in which immobilized proteins are used to capture binding partners from cell lysates. The novelty here is in the use of a complete, natural channel as bait to test for lipid organelle capture. Since non-specific binding is more of an issue in detergent-free PD experiments, results were limited to experiments where there was little binding to the untreated bead or immobilized rabbit IgG controls. Pre-blocked Ab571 controlled for the possibility that a protein other than the channel is attached to the beads and responsible for SV capture. We anticipate that the set of methods described herein will be highly utile for the analysis of SV-release site interactions – and indeed any vesicle–protein interaction. The four approaches discussed above are complementary and permit both the identification of a cell biological interaction and, ultimately, its analysis down to the level of specific amino acids.

We do not know if the channel binds directly to integral SV proteins or whether another protein(s) acts as a bridge. However, the finding that CaVs expressed in tsA201 cells can also capture SVs supports the idea that the interaction is between the channel and an integral or associated SV protein, and does not require an additional bridge protein specific to nerve terminals. Further, successful SV-PD using the C3_strep_ fusion protein supports involvement of this channel region and argues strongly for a direct cytoplasmic link. The molecular interaction proposed by Kaeser et al. (2011) is of particular interest. Their genetic analysis concludes that Rab3a on the SV binds to RIM acts as a bridge to the PDZ domain on the tip of the C-terminal (in addition to linking to the terminal via RBP2). An attractive feature of this model is that, based on reasonable estimates for disordered and predicted secondary structure, the distal end of the C-terminal may extend as far as 200 nm into the cytoplasm, providing a means to capture the SV from the nearby pool. Nonetheless, questions remain: first, we have repeatedly observed that while CaV2.2 and RIM co-vary at the release sites of intact terminals, the molecular interaction as assayed using biochemistry is weak (see also **Figure 5**, *CaV2.2 PD lane*). To explain this anomaly we suggested that RIM is involved in a switchable link (Wong and Stanley, 2010). A key role for the channel C-terminal in SV tethering may guarantee a maximum 1:1 ratio of these entities as suggested recently (Xue et al., 2012). CaV2.2 is the predominant presynaptic channel in chick but in mammals CaV2.1 plays a more important role. Thus, we would predict that the SV binding method would be conserved between these channels. The C-terminals exhibit highly conserved regions, including the tip PDZ-ligand domain (PDZ-LD). Further analysis of the molecular mechanism of tethering, as assessed by the SV-PD method, will be the subject of a future report.

It should be noted that while our findings support a direct C-terminal-based SV tether, they do not rule out other potential channel–SV tethering interactions. Indeed, additional links can be predicted based on estimates of inter-channel-SV distance estimates using morphological (Heuser et al., 1974; Stanley, 1997; Harlow et al., 2001; Stanley et al., 2003) or functional (Stanley, 1993; Mulligan et al., 2001; Weber et al., 2010) analyses which locate the calcium sensor within ~25 nm of the calcium channel pore. This is considerably shorter than even a conservative prediction of the length of a 350-amino acid channel C-terminal. A second attachment site could also explain how, in a two-armed scaffold model (Wong and Stanley, 2010), the PDZ-LD could both tether SVs while also contributing to channel scaffolding within the release site (Han et al., 2011; Kaeser et al., 2011). The II–III loop (Catterall, 1999) is a candidate a “secondary” tether mechanism. Channel–SV interactions may involve a complex molecular sequence that contributes to several steps in the docking-fusion cycle, consistent with imaging of SV-membrane links as imaged by EM tomography (Szule et al., 2012). We hypothesize that, considering its length, contact with the distal tip of the C-terminal may represent the link in which the channel captures the SV from the cytoplasm. Future studies will analyze the mechanism whereby the channel C-terminal binds to the SV and will search for additional SV tethering mechanisms.

## Conflict of Interest Statement

The authors declare that the research was conducted in the absence of any commercial or financial relationships that could be construed as a potential conflict of interest.
